# Report of the Sanitary Commission Appointed by the Common Council of New Orleans to Investigate the Causes of Disease in That Locality

**Published:** 1855-01

**Authors:** 


### EDITORIAL DEPARTMENT.

---

### *Report of the Sanitary Commission appointed by the Common Council of New Orleans to investigate the Causes of Disease in that locality.*

This Report so long and anxiously expected by the student of Etiology, is not yet through the press. Great expectations have been raised from the liberal provision made for it, from the scope of discussion allowed, and particularly from the high character of the gentlemen appointed to prepare it. At the head of the Commission was Prof. Edward H. Barton, of the University of Louisiana, who has better claims to distinction as a meteorologist, than any other man in the country, when we take into consideration the time through which his observations have extended, and their care, accuracy, and daily frequency.

Although the New Orleans printers get along very slowly with the Report, we are indebted to Prof. Barton for some of the proof sheets, which give us an inkling of its character, extent, and objects. If we do wrong in publishing a part of it here in Buffalo before its issue in New Orleans, Dr. Barton must accuse his own personal courtesy and kindness to us as the cause of the malfeasance.

After recounting the sources from which their data were derived, the commission give the following summary of the opinions at which they arrive:

"Out of these data, together with the experience of many years, have grown the materials which form the opinions and principles put forth in the Report, as to the origin and cause of yellow fever, of which no more may be said at present, than that two of these opinions and principles will be found of inestimable value after experiment and experience shall have fully attested their soundness and infallibility.

"The one is, that yellow fever is, and always has been, here and elsewhere, a *preventable disease*, and

"The other is, that the presence of two general hygienic conditions are absolutely indispensable to the organization and transmission of the disease —the one of them, atmospheric—the other, terrene. These must meet *in*

*combination*, or there will be no result. The absence of *one*, as to *this* disease, is as the absence of *both*, and as one of these conditions is almost wholly within the control of man, and the other partially so, it must follow that his power extends to the prevention and expulsion of *this* disease.

"These sources too—the facts and the principles, constitute the bases of all the preventive and remedial measures with which the Report closes, and which were specially devised for practical execution through the ministrations of the city authorities.

"Throughout the several Reports we have constantly endeavored to avoid speculative opinions—to adapt all our principles and suggestions to practical ends, and having the great object of our appointment—utility to our stricken city—ever before our eyes—it has been to us as a polar star for our guidance.

"With the presentation of this Report, the authority of the commission ceases. Its labors and its functions end together, yet its members cannot part with the voluminous record of their toils, without an expression of their entire and unwavering confidence, that, if the preventive and remedial measures they have recommended, shall be fully carried out, rigidly enforced and perseveringly maintained by the city authorities—it would be altogether impossible for the yellow fever to originate here, or to be disseminated as an epidemic, if brought here from abroad."

The Commission was made up of Hon. A. D. Crossman, mayor of the city, Drs. E. H. Barton, A. F. Axson, S. D. McNeil, J. C. Simonds, and J. L. Riddell.

"To this Commission was deputed special instructions for inquiry and investigation, viz:

"1st. To inquire into the origin and mode of transmission or propagation of the late epidemic, yellow fever.

"2d. To inquire into the subject of sewerage and common drains, their adaptability to the situation of our city, and their influence on health.

"3d. To inquire into the subject of quarantine, its uses and applicability here, and its influence in protecting the city from epidemic and contagious maladies, and

"4th. To make a thorough examination into the sanitary condition of the city, into all causes influencing it in present and previous years, and to suggest the requisite sanitary measures to remove or prevent them, and into the causes of yellow fever in ports and other localities having intercourse with New Orleans."

After a tribute to the zeal and courtesy of the numerous gentlemen both in North and South America, who had contributed to the materials of the Commission, and recounting the general importance of sanitary and hygienic measures, they assign the virulence of the epidemics to their causes. The importance and availability of some of their deductions will at once attract notice.

"The *causes* of this insalubrity have been most carefully scrutinized, and it is our deliberate and gratifying conviction that they are fairly ascribable to local conditions which are mainly removable. A reference to some of them here, the principles applicable to them, and the recommendation for their removal or abatement, will not be inapplicable in anticipation of the Reports themselves.

"Throughout the vast period to which this investigation has extended, commencing when the population did not exceed 8,756, (in 1796,) *no epidemic* has occurred that has not been preceded and accompanied by a great disturbance of the original soil of the country, (in digging and clearing out canals, basins, &c.,) although other local causes doubtless had their influence. This has been so unequivocal and so constant, and *without an exception*, that it seems to the Commission to bear the relation of cause and effect. The proofs of it are furnished in the following pages, and might have been greatly extended in its more local influences. This disturbance seems to have generally taken place with great recklessness, manifestly preferring for the purpose the warm season, during which it is most dangerous.

"We have to make the same remark in relation to the clearing and draining the neighboring swamps, both of vast public utility, and when done in a suitable season and proper manner, under enlightened advisement, not injurious to the public health; but most disastrous, when these are not faithfully observed, as medical annals for hundreds of years past fully attest.

"The numerous undrained unfilled lots and squares dotting the surface of the city, becoming muddy pools in the rainy, which is always the sickly season, and common receptacles for filth and garbage of all kinds, are exhibited in our *sanitary map*, and should be early abated."

Besides these, others of the frequent causes of disease in cities are enumerated: such as defective sewerage, privies, street cleaning, wharves, &c. The Commission, after premising that the city has causes enough within itself to account for its insalubrity, recommend in relation to the subject of *quarantine* that "although the Commission is unanimous in the belief that no system, however rigid or successfully carried out, can ever be a substitute for the sanitary or preventive measures we have recommended, and which if properly enforced, would be at once a protection against both the origination and spread of yellow fever and cholera among us, yet in the imperfection that must attach to all such measures, we unite in recommending the establishment of a quarantine below the city, under the surveillance and control of the 'Health Department,' thereby preventing *all foul vessels* from entering the port with *diseased passengers or crew*, *placing the restriction only where it is a measure of safety*, and furnishing character and security abroad to our intercourse with other nations."

As to the origination and communicability of yellow fever, "the conclusion we have come to is, that yellow fever is not a disease personally contagious, that its infectious properties are only communicable in a foul or infectious atmosphere; that is, that a foul vessel or individual with the disease, will only propagate it, under atmospherical and local conditions similar to that which furnished its nativity. That although vitiated or infectious air may be conveyed in goods and in various ways to distant places, ventilation speedily dissipates it, and that if disease results, when it is much concentrated, or with very susceptible individuals, it extends no farther, except under the conditions above specified. * * *

* * * "Most careful scrutiny in the actual occurrences of the first eruption of the fever, its spread, the character of its localization, the subjects most liable and suffering from whatever class and country, have converted presumptive proof* into positive certainty, that the fever originated with us, that its fatal malignity and spread was justly attributable to a very remarkable concurrence and combination of atmospheric and terrene causes, always peculiarly fatal to human health and life. These have been most amply examined and fully pointed out, and the gratifying fact is shown, that at least one of these causes is entirely under our control, and that it is in our power greatly to diminish the other, and hence by *disseverance*, the fatal union is prevented."

We quote at length the conclusions as to the influence of meteorological causes:

"The results we have come to, after a careful analysis of the records in this climate, at least, during the several years through which these are reliable, and they have been made with great minuteness during the last twenty-one years, and corroborated as far as they go by those of every epidemic yellow fever and cholera that has existed in this country, of which there are any records; the more special details embracing nine epidemics of yellow fever and six of cholera, are embraced in the following table.

---

* Messrs. Riddell and Simonds dissent from the *positive* character of the proof.

"This table shows what an examination of the details of which it is but the concentrated result would more than justify.

"1. What are the several meteorological conditions of yellow fever and cholera at the *commencement, maximum intensity and declination* of these two diseases when existing in their *epidemic* grades.

"2. In comparison, it shows that cholera exists in a greater range of temperature and humidity than yellow fever.

"3. That these diversities constitute the pabulum for its support, so far as the mere climatic condition is concerned.

"4. That a higher solar radiation and atmospheric pressure exists during yellow fever periods than during cholera. Although the atmospheric pressure under which these two diseases prevail are shown by this average table to be about the same, the barometer continuing at a permanently higher grade, more regularly and constantly in yellow fever than in cholera, yet in this latter the fluctuations are much greater; indeed, it is so under all its climatic relations, as is abundantly shown in the large detailed table too extensive for this summary, of which this is a very condensed abstract.

"5. That for the existence of yellow fever a higher range of temperature and of dew-point for its commencement and maximum intensity, and that a declension of the former (temp.) to less than 70°, and the latter (dew-point) to near 60° puts a speedy end to its *epidemic* existence.

"6. That a larger quantity of rain usually falls, on an average, during the existence of yellow fever than during cholera.

"7. The 'drying power' is more variable during cholera than during yellow fever.

"8. The average duration of *epidemic* yellow fever has been 58.33 days, and the period of its influence decreasing, while the average duration of *cholera* has been 37.66 days, and the period increasing.

"These experiments are fully borne out by what we see daily verified of the ravages of these two very different diseases in the various climates that have been subject to them.

"If subsequent observations shall prove the correctness of these statements, the future occurrence and continuance of *epidemic* yellow fever will be ascertained with great probability by referring to a well kept meteorological register; it will show what valuable information is to be derived from connecting accurate and extensive meteorological experiments with the Health Department, recommended in a subsequent report.

"There are but two practical remarks which we deem it necessary to draw from this table, and from the showing in the reports: the first is, that although it is easier to keep free of yellow fever than cholera, we can exercise much influence on the causation of both, even in their climatic relations; and secondly, the *combination* of the terrene and meteorological conditions which is *absolutely essential* to the existence of either, we *certainly* have it in our power greatly to control, because, by proper policing and regard to other hygienic measures, that condition is clearly under our influence."

We have already occupied so much space in the consideration of these leading principles of Dr. Barton's report, that we feel that we have anticipated somewhat the office of the reviewer. During the passage of this notice through the press, the entire report has reached us by mail, and will demand another examination.

One thing we may say here, at the risk of repetition hereafter. There is no one-idea-ism, no hobby-riding about this production. In assigning to terrene and atmospheric causes their proper share in the causation of disease, we find neither the exclusive doctrines of the miasmatists, nor the absurdity of those who, recognizing the weak points of the malarial hypothesis, fly wildly to the other extreme, and claim to find in the filth and foul odors of a dirty city its protection and safeguard against the ravages of epidemics.

The middle ground is the safest, and to use Dr. Barton's expressive phrase, we have, in the meteorological and terrene causes, the two blades of a pair of shears, useless when single, but the very shears of Atropos when combined.

We conclude our copious abstraction, (pillage?) of Dr. Barton's report, with the following table, to which we prefix an extract from a private letter from its distinguished author.

"The meteorological conditions in relation to it, (yellow fever,) are more definite and precise, and the extremes under which it prevails much more limited than those of cholera. I should like much to show you the immense tables I have been compelled to construct to obtain the little concentrated table of two lines, the averages of so many years."

In conclusion, we wish to acknowledge our indebtedness to Dr. Barton for his kindness in forwarding these proof-sheets, as well as for the valuable letters we have from time to time received from him relative to meteorological investigation.

The report of this Commission will, in itself, be a monument of Dr. Barton's untiring industry and devotion to the public good. Should his suggestions and recommendations be faithfully carried out, he will have a nobler monument in the health and prosperity of the great city of which he is so valuable a citizen. H.

